# Whole Genome Sequencing of Extended Spectrum β-lactamase (ESBL)-producing *Klebsiella pneumoniae* Isolated from Hospitalized Patients in KwaZulu-Natal, South Africa

**DOI:** 10.1038/s41598-019-42672-2

**Published:** 2019-04-18

**Authors:** Raspail Carrel Founou, Luria Leslie Founou, Mushal Allam, Arshad Ismail, Sabiha Yusuf Essack

**Affiliations:** 1 Antimicrobial Research Unit, College of Health Sciences, University of KwaZulu-Natal, Durban, South Africa; 2 Department of Clinical Microbiology, Centre of Expertise and Biological Diagnostic of Cameroon (CEDBCAM), Yaoundé, Cameroon; 3 Department of Food Safety and Environmental Microbiology, Centre of Expertise and Biological Diagnostic of Cameroon, Yaoundé, Cameroon; 4 Sequencing Core Facility, National Health Laboratory Service, Johannesburg, South Africa

**Keywords:** Microbial genetics, Microbial genetics, Clinical microbiology, Clinical microbiology

## Abstract

Extended spectrum β-lactamase (ESBL)-producing *Klebsiella pneumoniae* remain a critical clinical concern worldwide. The aim of this study was to characterize ESBL-producing *K. pneumoniae* detected within and between two hospitals in uMgungundlovu district, South Africa, using whole genome sequencing (WGS). An observational period prevalence study on antibiotic-resistant ESKAPE (i.e. *Enterococcus faecium*, *Staphylococcus aureus*, *Klebsiella pneumoniae*, *Acinetobacter baumannii*, *Pseudomonas aeruginosa*, *Enterobacter spp*.) bacteria was carried out in hospitalized patients during a two-month period in 2017. Rectal swabs and clinical specimens were collected from patients hospitalized and were screened for ESBL-producing, Gram-negative ESKAPE bacteria using cefotaxime-containing MacConkey agar and ESBL combination disk tests. Nine confirmed ESBL-*K. pneumoniae* isolated from six patients and two hospitals were whole genome sequenced using an Illumina MiSeq platform. Genome sequences were screened for presence of integrons, insertion sequences, plasmid replicons, CRISPR regions, resistance genes and virulence genes using different software tools. Of the 159 resistant Gram-negative isolates collected, 31 (19.50%) were ESBL-producers, of which, nine (29.03%) were ESBL-*K. pneumoniae*. The nine *K. pneumoniae* isolates harboured several β-lactamase genes, including *bla*_CTX-M-15_, *bla*_TEM-1b_, *bla*_SHV-1_, *bla*_OXA-1_ concomitantly with many other resistance genes e.g. *acc*(6′)-lb-cr, *aad*AI6, *oqx*A and *oqx*B that confer resistance to aminoglycosides and/or fluoroquinolones, respectively. Three replicon plasmid types were detected in both clinical and carriage isolates, namely ColRNAI, IncFIB(K), IncF(II). Sequence type ST152 was confirmed in two patients (one carriage isolate detected on admission and one isolate implicated in infection) in one hospital. In contrast, ST983 was confirmed in a clinical and a carriage isolate of two patients in two different hospitals. Our data indicate introduction of ESBL-producing *K. pneumoniae* isolates into hospitals from the community. We also found evidence of nosocomial transmission within a hospital and transmission between different hospitals. The Clustered Regularly Interspaced Palindromic Repeats (CRISPR)-associated *cas*3 genes were further detected in two of the nine ESBL-KP isolates. This study showed that both district and tertiary hospital in uMgungundlovu District were reservoirs for several resistance determinants and highlighted the necessity to efficiently and routinely screen patients, particularly those receiving extensive antibiotic treatment and long-term hospitalization stay. It also reinforced the importance of infection, prevention and control measures to reduce the dissemination of antibiotic resistance within the hospital referral system in this district.

## Introduction

The emergence of extended-spectrum-β-lactamases (ESBLs)-producing *Klebsiella pneumoniae* (ESBL-KP) represent a serious clinical concern in both healthcare and community settings^1–3^. ESBL-producing *Enterobacteriaceae* including *K. pneumoniae* were listed as pathogens of critical priority for research and development of antibiotics by the World Health Organization in 2017^4^.

ESBL enzymes confer resistance to a large spectrum of β-lactam antibiotics including penicillins and third-generation cephalosporins, thus limiting effective therapeutic options, increasing morbidity, mortality and hospital costs^2,5,6^. Among the four molecular classes of beta-lactamases (A, B, C, and D) defined according to the Ambler classification, the most prevalent ESBLs belong to class A, namely CTX-M, TEM and SHV families. CTX-M enzymes are predominant worldwide; and five different groups of CTX-M including CTX-M-1-2-8-9-25 groups have been described in *K. pneumoniae* strains^1,2,7^.

A group of clinical bacteria, termed “ESKAPE” (*Enterococcus spp*., *Staphylococcus aureus*, *Klebsiella pneumoniae*, *Acinetobacter baumannii*, *Pseudomonas aeruginosa* and *Enterobacter spp*.) pathogens encompass bacteria that can escape the activity of and develop high levels of resistance to multiple antibiotics^8^. A study on the complexity and diversity of 25 *K. pneumoniae* strains isolated in clinical samples (sputum, endotracheal aspiration, blood, pleural cavity tap, urine) showed different resistance genes with 100% prevalence of *bla*_TEM_ and *bla*_SHV_. Additionally_,_ various TEM and SHV types (SHV-19, 20, 21 and 22, and TEM-1, 53 and 63) were observed in *K. pneumoniae* isolates in KwaZulu-Natal^9^. Similarly, several studies conducted in the Western Cape region of South Africa have shown that various TEM-, SHV-, and CTX-M types were commonly detected in South African clinical isolates of *K. pneumoniae*^10,11^. The plasticity of *K. pneumoniae* related to the acquisition of resistance genes coupled with its propensity to act as nosocomial pathogen underscore the necessity for further investigation in different South African settings. This study undertook the molecular characterization of antibiotic resistance genes, virulence factors and mobile genetic elements associated with ESBL-KP isolated from carriage and clinical samples from patients in the public hospitals in the uMgungundlovu district, KwaZulu-Natal, South Africa using whole genome sequencing (WGS).

## Results

### Bacterial strains and phenotypic analyses

Of 159 cefotaxime-resistant Gram-negative ESKAPE bacteria isolated, 31 (19.5%) were ESBL-producers including *K. pneumoniae* (n = 9, 29.03%), *Acinetobacter baumannii* (n = 8, 25.81%), *Pseudomonas aeruginosa* (n = 8, 25.81%), *Enterobacter aerogenes* (n = 4, 12.90%) and *Enterobacter cloacae* (n = 2, 6.45%). Only the nine *K. pneumoniae* isolated from six patients were further analyzed. Amongst them, three (33%) were clinical isolates identified in the tertiary hospital and the remaining six (67%) isolates were identified in rectal swabs in both district (n = 3) and tertiary (n = 3) hospitals. Table 1 shows antibiotic resistance profiles of these isolates. All nine ESBL *K. pneumoniae* isolates were resistant to penicillins and cephalosporins, aminoglycosides, fluoroquinolones and trimethoprim (Table 1). The resistance observed was corroborated with WGS analyses which revealed the presence of several antimicrobial resistance determinants.

**Table 1:** Antibiotic resistance profiles of carriage and clinical ESBL-producing *K. pneumoniae* isolates.

Isolate ID	Patient ID (date of isolation)	MLST	Hospital	MIC values (µg/ml)										
				Ampicillin	Cefoxitin	Cefotaxime	Ceftazidime	Imipenem	Meropenem	Gentamicin	Amikacin	Ciprofloxacin	Ofloxacin	Trimethoprim
**Carriage isolates**														
A105R2B2	A105 (10/06/2017)	ST607	District	≥512	≥512	≥512	≥512	16	16	16	32	64	128	≥512
A105R1B5		ST983	District	≥512	≥512	≥512	≥512	32	16	8	8	64	128	≥512
A111R1B2	A111 (10/06/2017)	ST17	District	≥512	≥512	≥512	32	2	0.25	4	8	32	64	≥512
G702R1B5	G702 (01/07/2017)	ST152	Tertiary	≥512	64	≥512	≥512	8	0.5	128	32	≥512	≥512	≥512
G702R2B5	G702 (03/07/2017)	ST152	Tertiary	≥512	≥512	≥512	≥512	4	2	≥512	128	≥512	≥512	≥512
G702R3B2	G702 (04/07/2017)	ST152	Tertiary	≥512	8	≥512	≥512	64	0.5	512	8	64	64	≥512
**Clinical isolates**														
ED01500733	ED01500733 (13/07/2017)	ST983	Tertiary	≥32	≤4	≥64	16	≤0.25	≤0.25	≥16	≤2	2	2	≥512
ED01502268	ED01502268 (17/07/2017)	ST432	Tertiary	≥32	≤4	≥64	≥64	≤0.25	≤0.25	16	8	4	8	≥512
ED01503757	ED01503757 (23/07/2017)	ST152	Tertiary	≥32	≤4	≥64	≥64	≤0.25	≤0.25	≥16	8	4	4	≥512

Moreover, three isolates from three different patients showed imipenem resistance while meropenem resistance was observed in two isolates and elevated MICs for imipenem were evident in several isolates. The MICs of carbapenems varied across the type of specimen but all carriage isolates except A111R1B2 were resistant to imipenem according to the EUCAST breakpoints. In addition, two isolates (A105R1B5 and A105R2B2) out of the six carriage *K. pneumoniae* were resistant to meropenem. Carbapenemase genes were not found in the nine *K. pneumoniae* isolates although several isolates were resistant to imipenem and meropenem or evidenced elevated carbapenem MICs. Previous studies showed that deficiency or alteration of OmpK35 and OmpK36 confers increased resistance to carbapenems *K. pneumoniae*^12,13^. OMP analysis subsequently revealed the absence of OmpK35 and OmpK36 genes in all *K. pneumoniae* isolates although the wild type OmpK37 gene was present.

### Genotypic analyses and antimicrobial resistance determinants

The nine ESBL-*K. pneumoniae* isolates harbored several resistance genes conferring resistance to aminoglycosides [*aad*AI6, *aac*(6′)Ib-cr, *aac*(3′)II-a, *aph*(6)Id, *aph*(3′)-Ib], sulphamides (*sul*1, *sul*2), tetracyclines (*tet*A, *tet*B, *tet*D), trimethroprim (*dfr*A14, *dfr*A27), phenicols (*cat*A1, *cat*B4) and fluoroquinolones (*oqx*A, *oqx*B). The most common β-lactam resistance genes were *bla*_TEM-1-B_ (n = 9; 100%), *bla*_CTX-M-15_ (n = 9; 100%), *bla*_SHV-1_ (n = 8; 89%) and *bla*_OXA-1_ (n = 4; 44.5%). In addition, *fos*A (n = 9; 100%) and ARR-3 (n = 6; 66.7%) encoding for resistance to fosfomycin and rifampicin, respectively, were also detected. Alterations in the fluoroquinolone drug target due to modification in the quinolone-resistance-determining region (QRDR) including DNA gyrase subunit A (*gyr*A) and topoisomerase IV subunit C (*par*C) were observed in four isolates (ED01503757, G702R1B5, G702R2B5 and G702R3B2). In *gyr*A, mutation encoding the amino-acid substitution serine at codon 83 to phenyalanine (Ser83F) was observed in all four isolates whilst in parC the amino-acid substitution concerned serine at codon 80 to leucine (Ser80L). All *K. pneumoniae* strains carried an integron integrase gene IntIPac.

### Multi-drug resistant (MDR) efflux pumps

All *K. pneumoniae* isolates harbored numerous MDR efflux pump genes including CmeA, CmeB, MATE, MFS, MacA, MarcB, MarA, OML, RND, AcrB and AcrAB. These MDR efflux pumps encode for resistance to several families of antibiotics including tetracyclines, fluoroquinolones, macrolides, tigecycline and β-lactams.

### Multi-locus sequence type analysis (MLST) and core genome multi-locus sequence type analysis (cgMLST)

Analyses of MLST profiles has shown high variation among the seven housekeeping genes and identified five different sequence types (STs) including ST152 (n = 4), ST983 (n = 2), and three singleton ST432, ST607 and ST17 (Table 2). The four *K. pneumoniae* ST152 strains isolated from two patients were detected in clinical (n = 1) and carriage (n = 3) samples in the tertiary hospital while the two *K. pneumoniae* ST983 were each identified in carriage and clinical sample of patients admitted in the district and tertiary hospital, respectively (Table 2). The single-locus variants ST432, ST607 and ST17 were isolated from tertiary (n = 1) and district (n = 2) hospital, respectively.

**Table 2:** Summary of phenotypic and genotypic characteristics of ESBL-producing *K. pneumoniae* isolates.

Isolate	Patient ID (date of isolation)	Hospital levels	Sample types	*Antibiotic resistance genes	Plasmid types	pMLST	MLST	Integrons
A105R2B2	A105 (10/06/2017)	District	Carriage	*aad*AI6, *aad*AI6/*aad*A10, *aac*(6′)Ib-cr, *aac*(3)-IIa, *aac*(3)-IId, *aph*(6)Id, *bla*_SHV-1_, *bla*_SHV-1_, *bla*_TEM-1B_, *bla*_CTXM-15_, *oqx*A, *oqx*B, *fos*A, *qnrB6*, *ARR*-3, *sul*1, *sul*2, *dfr*A27	ColRNAI, FIA(pBK30683), IncFIB(K), FII(pBK30683), IncFII(K), IncR	IncF [K13:A13:B-]	ST607	IntIPac
A111R1B2	A111 (10/06/2017)	District	Carriage	*aad*AI6, *aad*A6/aadA10, *aac*(6′)Ib-cr, *aph*(6)Id, *aph*(3′)Ib, *aac*(3)-IIa, *bla*_TEM-1B_, *bla*_SHV-11_, *bla*_CTX-M-15_, *bla*_SCO-1_, *oqx*A, *oqx*B, *qnrB6*, *qnrB54, fos*A2, *ARR*-3, *sul*1, *sul*2, *dfr*A27	ColRNAI, FIA(pBK30683), IncFIB(K), IncFII(K), IncR	IncF [K2-like:A13:B-]	ST17	IntIPac
ED01502268	ED01502268 (17/07/2017)	Tertiary	Clinical	*aph*(6)Id, *aph*(3’)Ib, *bla*_TEM-1B_, *bla*_SHV-60_, *bla*_CTX-M-14_, *oqx*A, *oqx*B, *fos*A, *sul*1, *sul*2, *tet*(A), *tet*(C), dfrA1	IncFIB(K), IncFII(K)	IncF [K9:A-:B-]	ST432	IntIPac
ED01500733	ED01500733 (13/07/2017)	Tertiary	Clinical	aph(6)Id, aph(3′)Ib, *bla*_TEM-1B_, *bla*_SHV-38_, *bla*_SHV-168_, *bla*_CTX-M-15_, *qnrB6*, *oqx*A, *oqx*B, *fos*A, *sul*2, *tet*(A), *tet*(C), *dfr*A14, *qnr*B1	IncFIB(K), IncFII(K)	IncF [K7:A-:B-]	ST983	IntIPac
A105R1B5	A105 (10/06/2017)	District	Carriage	*aph*(6)Id, *aph*(3′)Ib, *bla*_*TEM-1B*_, *bla*_SHV-38_, *bla*_SHV-168,_ *bla*_CTX-M-15_, *qnrB6*, *qnrB1*, *fos*A2, *oqx*A, *oqx*B, *fos*A, *sul*2, *tet*(A), *tet*(C), *dfr*A14	IncFIB(K), IncFII(K)	IncF [K7:A-:B-]	ST983	IntIPac
G702R3B2	G702 (04/07/2017)	Tertiary	Carriage	*aad*AI6, *aac*(6′)Ib-cr, *aac*(3)-IIa, *aph*(6)-Id, *aph*(3′)-Ib,*bla*_TEM-1B_, *bla*_SHV-1_, *bla*_CTX-M-15_, *bla*_OXA-1_, *oqx*A, *oqx*B, *fos*A, *mph*(A), *cat*B4, *cat*A1, *ARR*-3, *sul*1, *sul*2, *tet*(D), *dfr*A27	ColRNAI, IncFIB(K), IncF(II)	IncF [K12:A-:B-]	ST152	IntIPac
ED01503757	ED01503757 (23/07/2017)	Tertiary	Clinical	*aad*AI, *aad*A2, *aad*AI6, *aad*AI6/aadA10, *aac*(6′)Ib-cr, *aac*(3)-IIa, *aph*(6)-Id, *aph*(3′)-Ib,*bla*_TEM-1B_, *bla*_SHV-1_, *bla*_CTX-M-15_, *bla*_OXA-1_, *oqx*A, *oqx*B, *fos*A, *mph*(A), *cat*B4, *cat*A1, *ARR*-3, *sul*1, *sul*2, *tet*(D), *tet*(C), *dfr*A27	ColRNAI, IncFIB(K), IncF(II)	IncF [K12:A-:B-]	ST152	IntIPac
G702R1B5	G702 (01/07/2017)	Tertiary	Carriage	*aad*AI6, *aad*AI6/aadA10, *aac*(6′)Ib-cr, *aac*(3)-IIa, *aph*(6)-Id, *aph*(3′)-Ib, *aac*(6′)Ib8, *bla*_TEM-1B_, *bla*_SHV-1_, *bla*_CTX-M-15_, *bla*_OXA-1_, *oqx*A, *oqx*B, *fos*A, *mph*(A), *cat*I, *cat*A1, *cat*B3, *ARR*-3, *sul*1, *sul*2, *dfr*A27, *tet*(C)	ColRNAI, IncFIB(K), IncF(II)	IncF [K12:A-:B-]	ST152	IntIPac
G702R2B5	G702 (03/07/2017)	Tertiary	Carriage	*aad*A5, *aad*AI6, *aad*AI6/aadA10, *aac*(6′)Ib8, *aac*(6′)Ib-cr, *aac*(3)-IIa, *aph*(6)-Id, *aph*(3′)-Ib, *bla*_TEM-1B_, *bla*_SHV-1_, *bla*_CTX-M-15_, *bla*_OXA-1_, *oqx*A, *oqx*B, *qnrB6*, *fos*A, *mph*(A), *cat*A1, *cat*I, *cat*B3, *cat*B4, *ARR*-3, *sul*1, *sul*2, *tet*(D), *tet*(A), *dfr*A27, *dfr*A14, *tet*(C)	ColRNAI, IncFIB(K), IncF(II), ColpVC, IncFIB(pKPHS1), IncFII(K), IncN, IncQ1	IncN ST-5IncF [K12:A-:B-]	ST152	IntIPac

*Altogether, 2605 antibiotic resistance genes were investigated; pMLST: Plasmid multi-locus sequence type.

**Figure 1:**
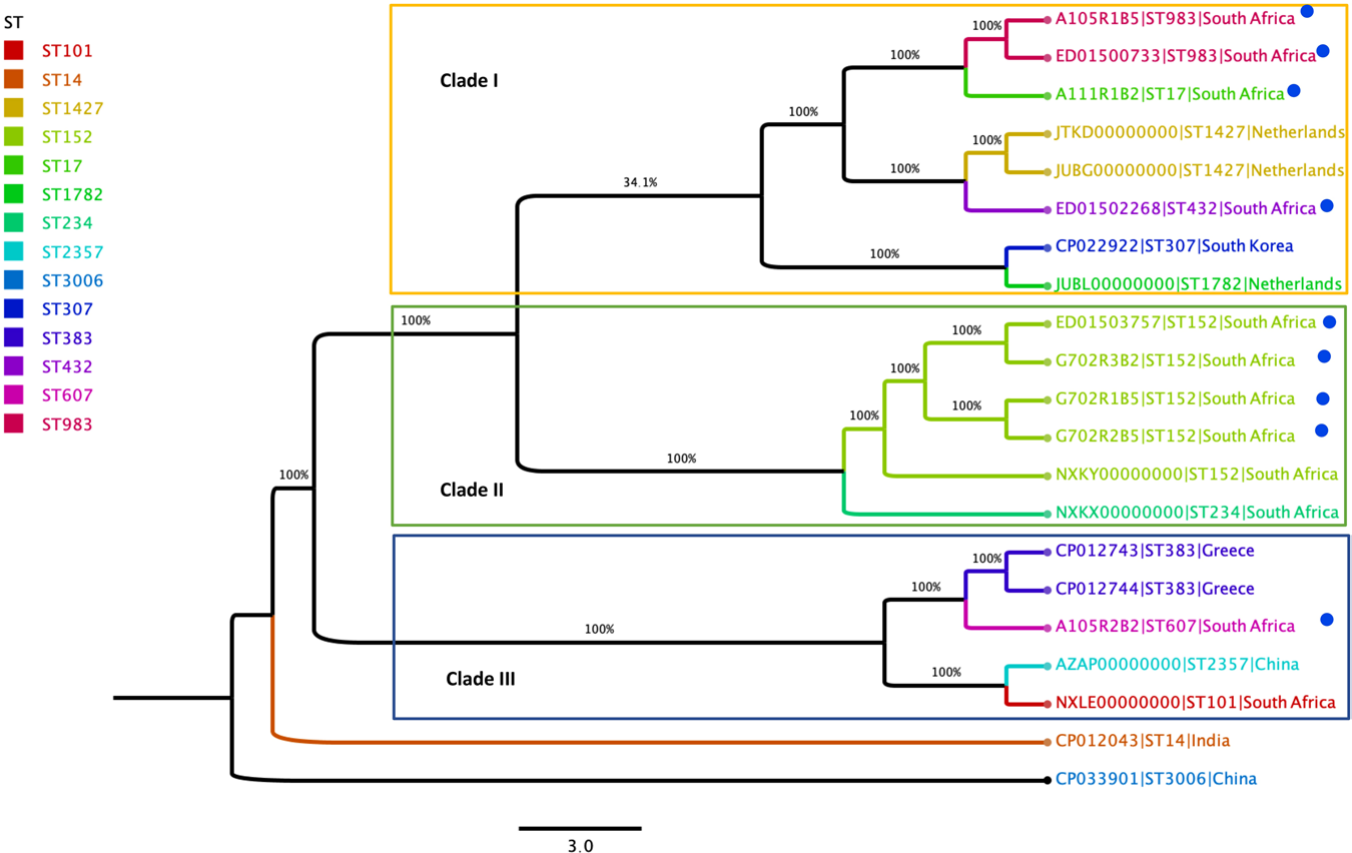
Phylogeny based on core genome multilocus sequence typing genes of 21 *K. pneumoniae* genomes. The following information is provided for each isolate: name/reference, **MLST types (STs)**, and country. STs are highlighted as indicated in the legend and isolates present in the study are marked with a blue dot.

The cgMLST *K. pneumoniae* scheme was defined with NCBI data using *K. pneumoniae* K069 as the reference genome. The close relatedness between a batch of carriage (A105R1B5) and clinical (ED01500733) ST 983 strains isolated from the district and tertiary hospital, respectively was evident, with 100% identity and an allelic distance of zero (Fig. 1). Similarly, high genetic similarity was observed between carriage (G702R3B2, G702R1B5, G702R2B5) and clinical *K. pneumoniae* (ED01503757) ST152 strains originating from the tertiary hospital with 99% identity and an allelic distance of zero (Fig. 1).

### Mobile genetic elements (MGEs) analysis

PlasmidFinder showed that all strains harboured at least two plasmids of the incompatibility group F (IncF), namely the IncFII and IncFIB (Table 2). It further revealed that the IncF plasmid detected in all ESBL-KP ST152 were associated with the same plasmid replicons ST, K12:A-:B-, while the ST of the IncF plasmid replicons detected in ESBL-KP ST983 isolates was K7:A-:B-. The carriage strains, A111R1B2 (ST17) and A105R1B2 (ST607) isolated in the district hospital, additionally carried an IncR plasmid. *In silico* plasmid analyses also revealed that one ESBL-KP ST152 (G702R2B5) isolated in a carriage sample harboured eight plasmids including ColpVC, ColRNAI, IncFIB(K), IncFIB(pKPHS1), IncFII, IncFII(K), IncN (ST-5) and IncQ1. In addition, an integron integrase IntIPac closely to IntI-1 was detected through ISFinder in all ESBL-KP strains (Table 2).

**Figure 2:**
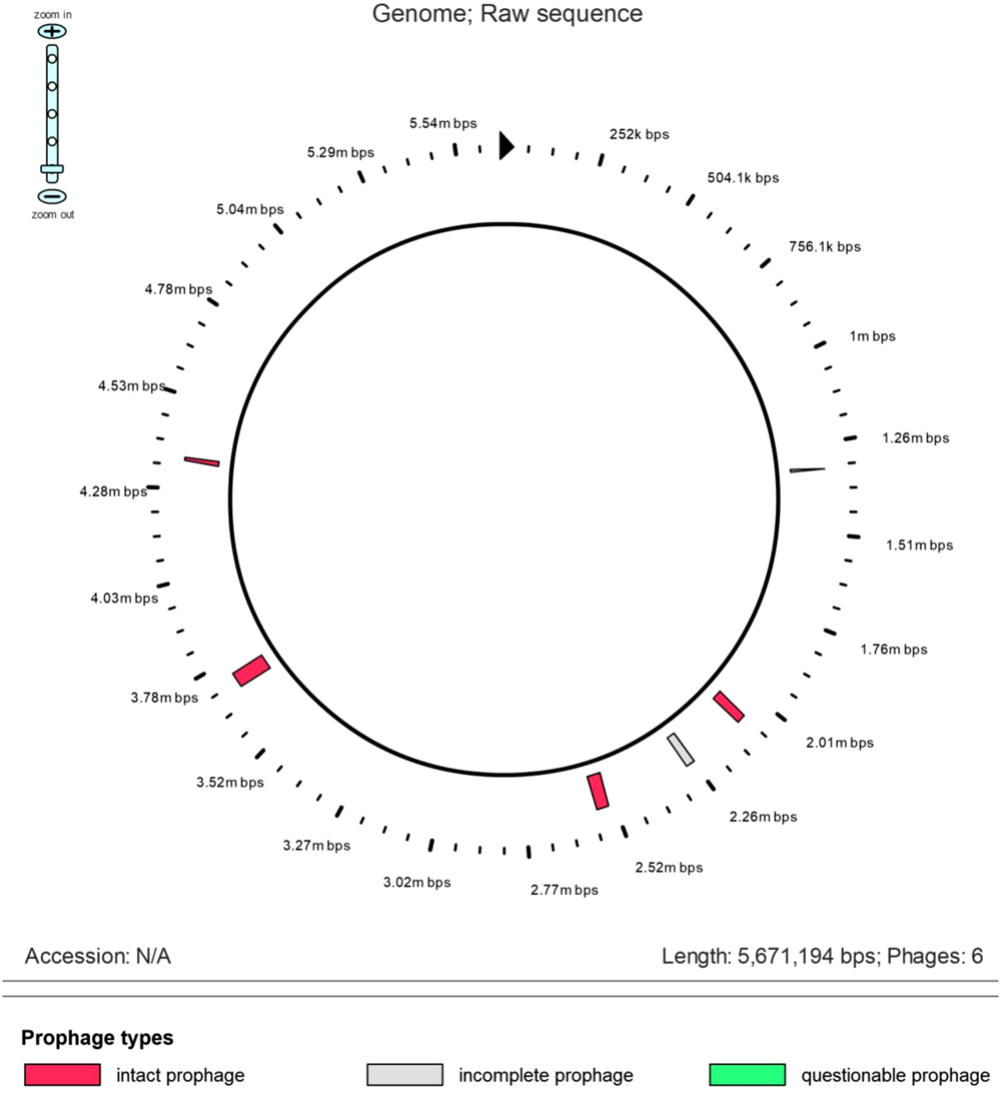
Genomic organization circular view of the phage regions in the *K. pneumoniae* ST152 (G702R2B5). The raw sequence presents the intact, incomplete and questionable prophage of *K. pneumoniae* G702R2B5.

**Figure 3:**
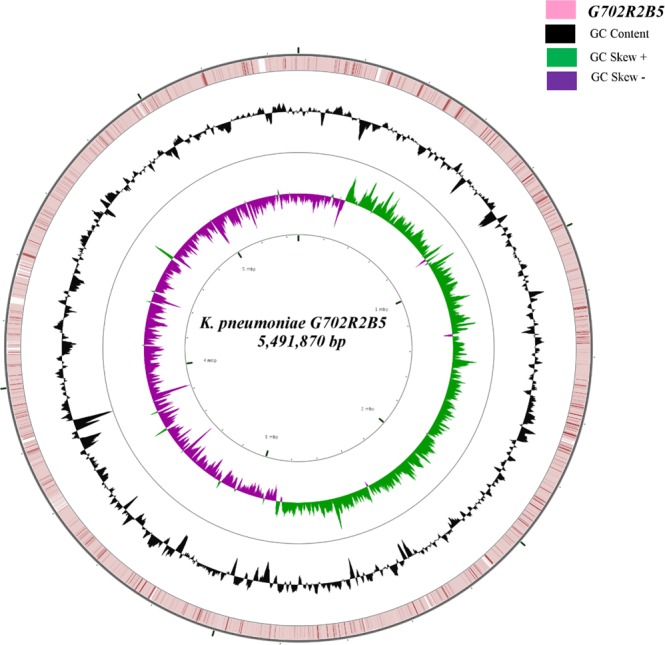
ESBL-producing *K. pneumoniae* G702R2B5 ring representation using CGView Server V 1.0^41^. The inner ring displays the percent of identity comparing the complete genome of *K. pneumoniae* U25(CP012043) and *K. pneumoniae* G702R2B5. The two next (inner) rings display the GC skew and GC content, respectively. The outer most ring indicates the complete genome *K. pneumoniae* G702R2B5.

PHAST algorithm demonstrated that 8 out of 9 strains (88.9%) hosted at least one intact bacteriophage (Supplementary Table 1). Entero P88 (n = 5; 62.5%) was the most prevalent intact phage followed by Salmon 118970 (n = 3; 37.5%), Entero mEp 235 (n = 2, 25%), and Klebsi phiKO2 (n = 2, 25%). Five and four phage regions were identified in *K. pneumoniae* ST152 (G702R2B5) and *K. pneumoniae* ST607 (A105R2B2) isolated in district hospital and carriage samples, respectively. Figure 2 shows the genomic organization of the phage regions in the *K. pneumoniae* ST152 (G702R2B5) while Fig. 3 represents its complete genomic structure.

**Figure 4:**
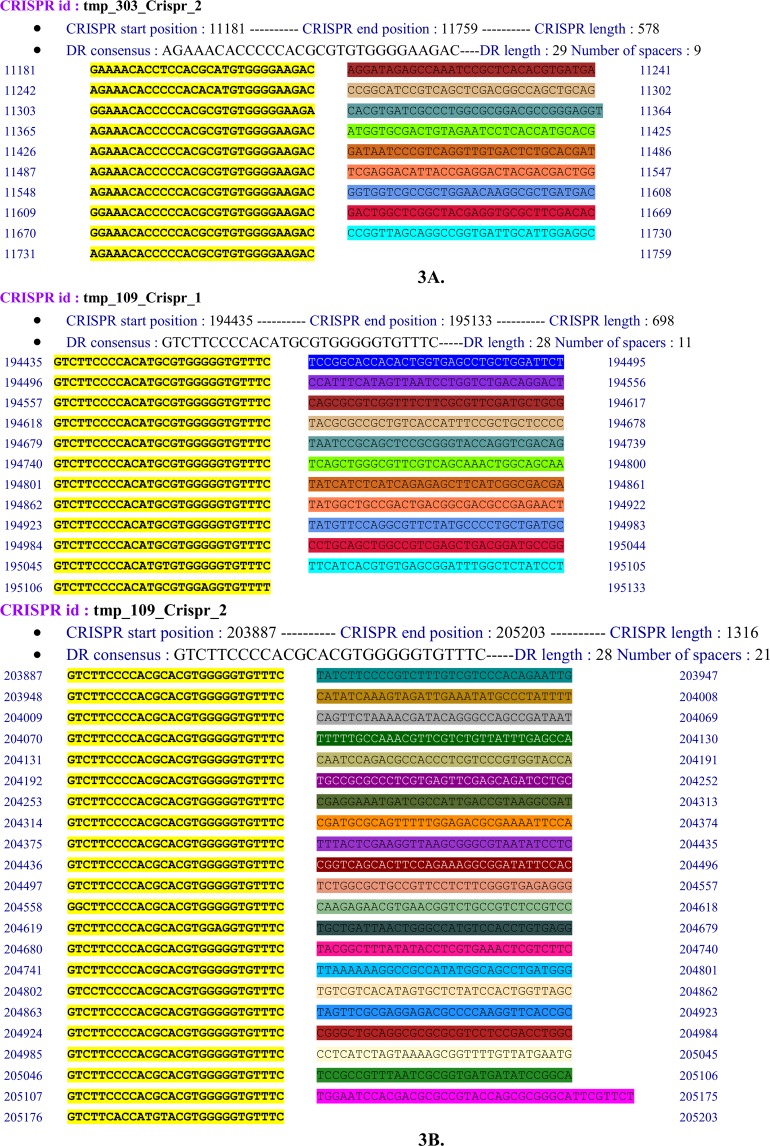
CRISP arrays detected in the *K. pneumoniae* G702R2B5 (3A) and A105R2B2 (3B). Two different Characterization of CRISPR arrays detected including (CRISPR 2) and (CRISPR 1 and 2) in *K. pneumoniae* G702R2B5 strain. The first CRISPR2 array composed of six direct repeated sequences and nine spacer sequences was located at nucleotides 11242 to 11731. CRISPR1 array composed twelve direct repeated sequences and eleven spacer sequences was located at nucleotides 194435 to 195045, CRISPR2 composed twenty-two direct repeated sequences and twenty-one spacers was located at nucleotides 203887 to 205176.

CRISPRFinder identified CRISPR (Clustered Regularly Interspaced Palindromic Repeats) regions in only 2 out of 9 ESBL-KP namely ESBL-KP ST607 (A105R2B2) and ST152 (G702R2B5). At least one CRISPR array including CRISPR1 or CRISPR2 was detected in these isolates. These two were both identified as CRISPR-associated *cas*3 gene (Fig. 4). ESBL-KP ST607 harboured six plasmids [ColRNAI, FIA(pBK30683), IncFIB(K), FII(pBK30683), IncFII(K) and IncR], four phages (Salmon SEN5, Entero P4, Pseudo JBD44, Entero fiAA91_ss) and both two CRISPRs (Supplementary Table 1). CRISPR1 and CRISPR2 were located at nucleotides 194435 to 195133 with 11 spacers and 203887 to 205203 with 21 spacers, respectively. In contrast, ESBL-KP ST152 (G702R2B5) carried eight plasmids [ColRNAI, IncFIB(K), IncFII, ColpVC, IncFIB(pKPHS1), IncFII(K), IncN and IncQ1], four phages (Klebsi PKP126, Salmon SSU5, Salmon SSU5, Salmon Fels 2) and the CRISPR1, located at nucleotides 11181 to 11759 with nine spacers.

## Discussion

The increasing prevalence of ESBL-KP remains a major clinical concern worldwide. To understand the molecular epidemiology of ESBL-KP, we studied using WGS, antibiotic resistance genes, MGEs and genetic lineages associated with circulating ESBL-KP isolated from carriage and clinical samples of hospitalized patients in uMgungundlovu district, South Africa.

The increasing prevalence of ESBL-KP has been associated with high mortality in developing country^14^. However, a 5% prevalence of ESBL-KP was detected in faecal carriage and clinical samples in our study. This is consistent with the report of Jallad *et al*.^15^, which shown 9.7% of ESBL-KP from faecal carriage among healthy patients in nursing homes in Lebanon^15^. In contrast, this finding is lower than that described by Perovic *et al*.^16^, where a 68.9% prevalence of ESBL-KP was detected in bloodstream infections in the public healthcare sector in Free State, Gauteng, Limpopo, KwaZulu-Natal, and Western Capes provinces in South Africa^16^. Similarly, Rashid *et al*.^5^ reported a 32.43% prevalence of ESBL-KP from faecal carriage in healthy patients hospitalized in tertiary hospital in India^5^. The discrepancies observed in the ESBL-KP prevalence could be attributed to variation of the geographic location, level of exposure to healthcare settings, hospital levels, antibiotic stewardship programs and antibiotic use.

The molecular characterization of diverse resistance determinants associated with the circulating ESBL-KP strains was undertaken following the health referral system. High level of resistance was detected in the tertiary hospital with one isolate, G702R2B5 (ST152) harboring 48 resistance genes in contrast to the district hospital where a maximum of 36 resistance genes were identified in the isolate A111R1B2 (ST17). The presence of genes encoding resistance to β-lactams, aminoglycosides, fluoroquinolones, fosfomycin, rifampicin, sulphonamide were reported in both clinical and carriage samples in the tertiary hospital. This is consonant to that reported in the literature where *bla*_CTX-M-15_, *bla*_SHV-28_, and *bla*_TEM-1B_ and *fos*A3 were the common genes implicated in the resistance of cephalosporins, monobactams and fosfomycin identified in carriage and clinical *K. pneumoniae* isolate in Lebanon^17^ and China^18^. This suggest that ESBL-KP either in clinical or carriage sample, could be a probable reservoir of resistance genes for other bacterial species and be responsible for genetic transfer to other species. The dissemination of ESBL-KP in these healthcare settings could probably be attributed to a lack of effective infection, prevention and control (IPC) measures for their containment.

An interesting finding of this study was the detection of the clonal lineage ST152 (n = 4; 44.5%) circulating in both carriage (at admission, n = 3) and clinical sample (n = 1) of the tertiary hospital. These isolates were characterized by their multidrug resistance which was confirmed by the concomitant presence of several β-lactam (*bla*_CTX-M-15_, *bla*_SHV-11_, *bla*_TEM-1B_ and *bla*_OXA-1_) resistance genes. This is consistent with the literature which confirmed that the *bla*_SHV_ gene is a normal chromosomal gene in *K. pneumoniae* and that CTX-M-15 is the most predominant CTX-M enzyme worldwide^19^. In addition, resistance genes for aminoglycoside [*aad*AI6, *aac*(6′)Ib-cr, *aac*(3)-IIa, *aph*(6)-Id and *aph*(3′)-Ib], fluoroquinolone (*oqx*A, *oqx*B, and *qnrB6*), fosfomycin (*fos*A), macrolide [*mph*(A)], rifampicin (*ARR*-3), phenicol (*cat*B4, *cat*A1), tetracycline [*tet*(D)], trimethoprim (*dfr*A27) and sulphonamide (*sul*1, *sul*2) were also identified in these isolates. It is acknowledged that *aac*(6′)-Ib-cr is a variant of the *aac*(6′)-Ib gene which acetylates fluoroquinolones and has a low-level resistance to aminoglycosides. Mutation in gyrA (Ser83F) and parC (Ser80L) have further been detected in these four ST 152 isolates. Our MICs corroborate these findings since all these isolates exhibited high level resistance to fluoroquinolones, except for *K. pneumoniae* ED01503757 where moderate fluoroquinolone resistance was observed. ESBL-KP harboring similar resistance genes have been reported in Italia^20^ and Lebanon hospital^8^. Tokajian *et al*.^17^, showed that CTX-M-15 was associated with MDR-*K. pneumoniae* and revealed that *qnr*B6 was frequently observed in African countries^17^. Several studies showed that ESBL-producing *K. pneumoniae* ST152 is associated with resistance to carbapenems^21–23^.

Taken all together, the fact that ESBL-KP ST152 strains isolated in the tertiary hospital harbored the same resistance genes and mobile genetic elements including plasmids [ColRNAI, IncFIB(K), IncF(II)] and integrons (IntIPac) suggests that this clone could be associated with intra- and/or inter-hospital dissemination. This clonal spread was corroborated in our cgMLST analysis, where in clade II a high genetic relationship (99.90% identity and allelic distance of zero), was observed in our collection of carriage (G702R3B2, G702R1B5, G702R2B5) and clinical *K. pneumoniae* (ED01503757) ST152 strains, all originating from the tertiary hospital. In addition, a close relationship was observed with the *K. pneumoniae* ST152 strain K069 (NXKY0000000) detected in a clinical sample in Pretoria, South Africa. Of further interest is that one of the *K. pneumoniae* ST152 (ED01503757) isolates was carbapenem susceptible whereas the other ST152 isolates showed reduced susceptibility to carbapenems. Although the contribution of efflux mechanisms was neither determined by a MIC reduction assay nor gene expression assay, we hypothesized that the overexpression or repression of the numerous multidrug resistance efflux pumps detected in these isolates could be associated with the various MICs of imipenem and meropenem as described^24^.

Another interesting finding of this study, was the detection of two ESBL-KP ST 983 harboring similar resistant determinants [*aph(*6)Id, *aph*(3’)Ib, *bla*_TEM-1B_, *bla*_SHV-38_, *bla*_CTX-M-15_, *qnrB6*6, *oqx*A, *oqx*B, *fos*A, *sul*2, *tet*(A), *dfr*A14], plasmids [IncFIB(K), IncFII(K)] and plasmid MLST [IncF (K7:A-:B-)]. They were isolated in two patients hospitalized in the district (A105R1B5) and tertiary (ED01500733) hospital, suggesting the probable clonal spread of the ESBL-KP ST983 inter-hospital in uMgungundlovu district as a result of the health referral system. The cgMLST analysis confirms that the two clinical (ED01500733) and carriage (A105R1B5) *K. pneumoniae* ST 983 in clade I were closely genetically related with 100% identity, an allelic distance of zero and an allele difference of one. Meanwhile, they shared a common ancestor with the *K. pneumoniae* ST 17 (A111R1B2) detected in the carriage sample of the district hospital. The two ST983 isolates ED01500733 and A105R1B5 were as such closely related to this ST17 isolate with an allelic distance of zero, a 99.41% percent identity and allele differences of 16640 and 16639 between A111R1B2 and A105R1B5, and between A111R1B2 and ED01500733, respectively. Our findings thus suggest that these ST 152 and ST 983 lineages could spread between patients in the same or different wards, within and/or across hospitals reflective of the referral system between hospitals in the public healthcare system. This spread further intimates sub-optimal infection prevention and control practices and provide motivation for the pro-active surveillance and routine screening of admitted patients in uMgungundlovu district in South Africa, in order to prevent the emergence and subsequent spread of new highly resistant clones.

Whilst Ambler classes A, B, C and D carbapenemase-producing *K. pneumoniae* strains gained worldwide attention due to the high resistance conferred to carbapenems, *K. pneumoniae* has evolved to become resistant to almost all β-lactams without harbouring carbapenemase genes^13^. This phenomenon has been possible with the concomitant use of multiple resistance mechanisms such as acquisition of an Ambler class A or C β-lactamases, with the loss of the OmpK35 and OmpK36 porins and/or overexpression of MDR efflux pumps^13^. In fact, some studies^25,26^ have established the impact of MDR-efflux pumps and porin losses on the membrane permeability of *K. pneumoniae*. The carbapenem resistance detected phenotypically (meropenem 16 mg/L; MIC imipenem 8–64 mg/L) in some isolates was not corroborated genotypically with the detection of specific carbapenemase encoding genes by WGS. This could hence be explained by the fact that resistance was not mediated by specific carbapenemase genes but rather by porin loss and/or MDR efflux pumps present in all isolates. Efflux systems have been reported in several clinically important bacteria and the overexpression of MDR efflux pumps can lead to high-level multi-drug resistance^25,26^. All the isolates harbored several MDR efflux pumps including CmeA, CmeB, MATE, MFS, MacA, MarcB, AcrB, MarA, OML, RND and AcrAB. We postulate that MDR efflux pumps were implicated in multi-drug resistance observed in the majority of isolates. *K. pneumoniae* contains three well-known porins including the two major porins OmpK35 and OmpK36 that are homologous to the OmpF and OmpC of *Escherichia coli* respectively, as well as the small porin OmpK37. Given that OmpK35 and OmpK36 porins play a critical role in the cell penetration of antibiotics, their loss can lead to reduce susceptibility or resistance to cephalosporins and carbapenems, especially in strains harboring Ambler class A, B, C or D β-lactamase^12,13^. The detection of OmpK37 porin that allows penetration by carbapenems but not other β-lactams, may explain why all isolates expressed high level resistance to cefoxitin (except for clinical isolates), cefotaxime and ceftazidime, and null or moderate resistance to carbapenems. We thus hypothesized that the deficiency in OmpK35 and OmpK36 coupled with the presence of *bla*_CTX-M-15_, and MDR efflux pumps, could play a significant role in conferring *K. pneumoniae* resistance to carbapenems and third generation cephalosporins in our study.

ABR is generally mediated by intrinsic or acquired resistance genes located on chromosome or MGEs, respectively. The CRISPR-Cas system was demonstrated to cleave plasmid DNA, thereby protecting bacteria from transduction (phage infection) and other horizontal gene transfers. They were supposed to be a defense mechanism against infection by diverse extra-chromosomal agents^26^. The correlation between the presence of CRISPR-Cas system and antibiotic resistance has already been studied and an inverse correlation between its presence and acquisition of antibiotic resistance was described in 48 *Enterococcus faecalis* strains^26^. In *K. pneumoniae*, CRISPR-Cas system has been detected in a very few strains worldwide. Apart from our isolates A105R2B2 and G702R2B5, only two complete *K. pneumoniae* genomes (NC_018522, NC_012731) and five draft genomes sequences (NC_012731, NZ_ANGH02000012, NZ_APGM01000001, NZ_JH930419, NZ_JH930428) harbor it. Even though CRISPR-Cas system serves to protect bacteria against phage infections and horizontal gene transfer, their presence among ESBL-KP ST607 (A105R2B2) and ST152 (G702R2B5) that were the most resistant isolates, suggest their probable implication in the acquisition of resistance genes. This is consistent with a report which demonstrated that in *Klebsiella* genomes, CRISPR-Cas systems are located among genes encoding for proteins that are likely involved in metabolism as well as resistance to antibiotics^22^. Additionally, the detection of several phages in these two highly resistant strains along with CRISPR-associated *cas*3 genes shed light on areas for further investigation on the emergence of ABR and transmission of antibiotic resistance genes.

In summary, our findings reveal the dissemination of ESBL-producing *K. pneumoniae* within and between wards and hospitals in uMgungundlovu district, South Africa. It shows that hospital is a reservoir for several resistance determinants and highlights the necessity to efficiently and routinely screen patients, particularly those receiving extensive antibiotic treatment and long-term hospitalization stay. It also reinforces the need for infection, prevention and control measures to reduce the dissemination of ABR in this district.

## Methods

### Ethical approval

Ethical approval was obtained from the Biomedical Research Ethics committee (BREC) (No. BF512/16, sub-study of BCA444/16) of the University of KwaZulu-Natal, South Africa. Permission to conduct the research was also granted from the Department of Health, uMgungundlovu District and hospital managers. All methods were performed in accordance with the relevant guidelines and regulations.

### Study design and bacterial isolates

This study took place in two hospitals at different level of care (district and tertiary), from May to July 2017 in uMgungundlovu district, South Africa. The district and tertiary hospitals were approximately 70 Km apart. Oral and written informed consent were obtained from all study participants after explanation of the procedure and purpose of the study. Rectal swabs were collected aseptically with Amies swabs from all admitted in-patients >18 years old to form the carriage sample. Isolates routinely processed in the microbiological laboratory during the sampling period formed the clinical sample. Every patient included in this study was screened for the presence of Gram-negative ESKAPE bacteria. All samples were cultured onto MacConkey agar with and without cefotaxime (2 mg/L). After incubation for 18–24 h at 37 °C, each morphotype growing on MacConkey with cefotaxime (MCA + CTX) was subjected to Gram staining, catalase and oxidase tests, followed by biochemical identification with API 20E (bioMérieux, Marcy l’Etoile, France) and the Vitek^®^ 2 System (bioMérieux, Marcy l’Etoile, France).

The strains sequenced in this study were isolated from carriage (A105R2B2, A105R1B2, A111R1B2, G702R1B5, G702R2B5, G702R3B2) and clinical samples including sputum (ED01503757, ED01502268) and urine (ED01500733) of six patients hospitalised in the district or tertiary hospital. The isolates A105R2B2, A105R1B2, A111R1B2 were detected in rectal swabs of two patients (A105 and A111) admitted in the medical ward of the district hospital while the isolates G702R1B5, G702R2B5, G702R3B2 were recovered from rectal swabs of a single patient (G702) but at different time-point (admission, after 48 h and at discharge) in the tertiary hospital. The clinical isolates ED01503757, ED01502268, ED01500733 were identified from three patients in the tertiary hospital. These isolates were closely related on enterobacterial-repetitive-polymerase chain reaction (ERIC-PCR)^27^ analysis^28^. Given that we aimed to evidence clonal spread of ESBL-KP, within each ERIC-cluster, representative isolates of related strains originating from different level of care were considered for WGS. These isolates were more representative because they belonged to the same ERIC-PCR cluster and antibiotic resistant patterns.

### Phenotypic screening of ESBL-production and antimicrobial susceptibility testing

All isolates were phenotypically screened for ESBL, AmpC, KPC and MBL production using combination disk test sets (ROSCO DIAGNOSTICA, Taastrup, Denmark). Minimum inhibitory concentrations (MICs) were determined broth microdilution for all selected isolates. Ampicillin, cefoxitin, cefuroxime, cefotaxime, ceftriaxone, imipenem, meropenem, amikacin, gentamicin, trimethoprim, ciprofloxacin, moxifloxacin, nitrofurantoin, tetracycline, were tested and interpreted according to the European Committee on Antimicrobial Susceptibility Testing (EUCAST) breakpoints^29^. *E. coli* ATCC 25922, *K. pneumoniae* ATCC 700603 and *K. pneumoniae* ATCC 51503 were used as controls.

### DNA Extraction

Genomic DNA (gDNA) was extracted with the GenElute® Bacterial Genomic DNA Kit (Sigma-Aldrich, St. Louis, MO, USA) according the manufacturer’s instructions. The purity and concentration of the extracted gDNA were determined by fluorometric analysis (Qubit®) and agarose gel electrophoresis.

### Genomic DNA Sequencing and assembly

Multiplexed paired-end libraries (2 × 300 bp) were prepared with the Nextera XT DNA sample preparation kit (Illumina, San Diego, CA, USA) and sequencing performed on an Illumina MiSeq instrument with 100 × coverage by the National Institute of Communicable Diseases Sequencing Core Facility, South Africa. The generated reads were quality trimmed and *de novo* assembled using CLC Genomics Workbench version 10 (CLC, Bio-QIAGEN, Aarhus, Denmark) and SPAdes version 3.5^30^ to nullify any gaps.

### Genome analysis

The assembled contigs were uploaded to the prokaryotic genome annotation pipeline server (https://www.ncbi.nlm.nih.gov/genome/annotation_prok/) and RAST server (http://rast.nmpdr.org/)^31^ for annotation.

### Identification of the resistome, virulome and mobile genetic elements

The GoSeqIt tool was used to annotate and determine known antimicrobial resistance genes, virulence factors and plasmids using ResFinder^32^, VirulenceFinder^33^ and PlasmidFinder^34^, respectively. The RAST SEED viewer aided the identification of integrons and transposases flanking the β-lactamase genes^35^. The identification, annotation and visualization of prophage associated regions were performed using PHAge Search Tool (PHAST) server^36^. Clustered Regularly Interspaced Short Palindromic Repeats (CRISPR) and insertion sequence elements were investigated with the CRISPRFinder server (http://crispr.i2bc.paris-saclay.fr/Server/) and ISFinder (https://www-is.biotoul.fr/)^37^, respectively. Outer membrane porin genes were analyzed with the Sequence Search Antibiotic Resistance Tool (SSTAR, version 1.1.01, https://github.com/tomdeman-bio/Sequence-Search-Tool-for-Antimicrobial-Resistance-SSTAR-)^38^ software that used a standalone Basic Local Alignment Search Tool (BLAST) and a database combining ARG-ANNOT and ResFinder to identify known antibiotic resistance genes, detect putative new variants, modification and/or truncated genes. In addition, the Comprehensive Antibiotic Resistance Database (CARD; https://card.mcmaster.ca) was used to corroborate the results. Finally, the contigs of the *K. pneumoniae* G702R2B5 were mapped against the complete genome of *K. pneumoniae* U25 (CP012043) for visualization of the genomic organization^39^.

### Multilocus Sequence typing (MLST) and core genome multi-locus sequence type analysis (cgMLST)

The scheme of Diancourt *et al*.^38^, which considers the allelic variation amongst seven housekeeping genes (*gapa*, *infb*, *mdh*, *pgi*, *phoe*, *rpob* and *tonb*) to assign STs was used for *in silico* multi-locus sequence type (MLST)-analyses and WGS data were used for the MLST assignment of *K. pneumoniae* isolates^40^.

A genome-wide gene-by-gene comparison approach was used to assess the clonal relatedness between isolates within and across wards and hospitals. The core genes were determined from the annotated genome assemblies, predicted coding regions were extracted and converted into protein sequences. A phylogeny was drawn for *K. pneumoniae* using Rapid large-scale prokaryote pangenome analysis (Roary; https://sanger-pathogens.github.io/Roary/) to estimate the tree for the core genome. The genome of *K. pneumoniae* strain K069 (Accession number NXKY01000005.1) served as reference genome and the following 12 query international *K. pneumoniae* genomes (Accession numbers JUBG00000000, JTKD00000000, JUBL00000000, JUBM00000000, AZAP00000000, CP012743, CP012744, CP012043, NXLE01000020.1, NXKY01000005.1, NXKX01000020.1, CP022922.1, CP033901.1) obtained from NCBI database were used to assess the cgMLST target genes. Altogether, 2944 core genes were extracted with an alignment length of 2,852,207 bp shared by the nine *K. pneumoniae* genomes. The allelic distance from the cgMLST was visualized using Figtree v1.4.3 (http://tree.bio.ed.ac.uk/software/figtree/) in a maximum likelihood phylogenetic tree including isolate name, ST type and country.

## Supplementary information

Table S1

## Data Availability

This whole-genome shotgun project PRJNA429538 of *K. pneumoniae* strains ED01500733, ED01502268, A105R2B2, A111R1B2, G702R3B2, ED01503757, A105R1B5, G702R1B5, G702R2B5 has been deposited at DDBJ/EMBL/GenBank under accession numbers POWS00000000, POTV00000000, POWR00000000, POTU00000000, POWQ00000000, POTT00000000, POTS00000000, POWP00000000 and POWO00000000, respectively.
