# Supplementary material for: Whole Genome Sequencing of Extended Spectrum β-lactamase (ESBL)-producing Klebsiella pneumoniae Isolated from Hospitalized Patients in KwaZulu-Natal, South Africa
Source: Sci Rep. 2019 Apr 18;9:6266. doi: 10.1038/s41598-019-42672-2 (PMC6472517; doi:10.1038/s41598-019-42672-2)
Supplement: Supplementary file 1 — Table S1 [file 41598_2019_42672_MOESM1_ESM.pdf]

Supplementary Information

Whole Genome Sequencing of Extended Spectrum β-lactamase (ESBL)-producing *Klebsiella pneumoniae* Isolated from Hospitalized Patients in KwaZulu-Natal, South Africa

Raspail Carrel Founou[1,2]*, Luria Leslie Founou[1,3], Mushal Allam[4], Arshad Ismail[4], Sabiha Yusuf Essack[1]

[1]Antimicrobial Research Unit, College of Health Sciences, University of KwaZulu-Natal, Durban, South Africa
[2]Department of Clinical Microbiology, Centre of Expertise and Biological Diagnostic of Cameroon (CEDBCAM), Yaoundé, Cameroon
[3]Department of Food Safety and Environmental Microbiology, Centre of Expertise and Biological Diagnostic of Cameroon, Yaoundé, Cameroon
[4]Sequencing Core Facility, National Health Laboratory Service, Johannesburg, South Africa

***Corresponding author:**

Raspail Carrel Founou

czangue@yahoo.fr

Tel: +237 675 94 35 67

Table S1. Distribution of intact prophage regions among the ESBL-producing *K. pneumoniae* strains. a: Intact prophage region; b: Region length of intact prophage; c: Phage with the highest number of CDS in the region and the number of gene counts in brackets.

Isolate name	ST	Region[a]	Length[b] (kb)	No CDS	GC%	Phage (hit genes count)[c]
**A105R2B2**	ST607	2	31.2	42	54.83	Salmon SEN5 (19)
		4	41.8	19	52.69	Entero P4 (5)
		5	48.6	45	51.90	Pseudo JBD44 (11)
		6	14.6	20	56.63	Entero fiAA91 ss (16)
**A111R1B2**	ST17	1	31.2	43	53.10	Salmon Fels 2 (32)
		7	47.1	42	53.33	Entero mEp235 (9)
		9	15.1	19	55.81	Entero P88 (12)
**A105R1B5**	ST983	1	60.1	44	52.80	Salmon 118970 sal4 (14)
**ED01500733**	ST983	2	57.2	45	53.62	Salmon 118970 (15)
		3	54.6	78	49.24	Klebsi JD001 (46)
**G702R3B2**	ST152	1	17.5	20	55.37	Entero P88 (12)
		2	30.4	33	50.48	Salmon 118970 (7)
		3	40.1	39	52.46	Klebsi phiKO2 (22)
**ED01503757**	ST152	1	38.1	38	53.37	Entero mEp235 (8)
		2	15.1	19	55.80	Entero P88 (11)
		3	40.7	31	52.84	Klebsi phiKO2 (20)
**G702R1B5**	ST152	1	22.1	26	52.68	Entero P88 (10)
		2	33.1	39	51.39	Bacill SP 15 (10)
		3	30.1	15	54.19	Entero P88 (6)
**G702R2B5**	ST152	1	43.4	51	51.99	Klebsi PKP126 (9)
		2	63.3	34	50.90	Salmon SSU5 (19)
		3	23.3	33	50.32	Salmon SSU5 (11)
		4	23.2	29	54.61	Salmon Fels 2 (22)
